# Supplementary material for: Content-rich biological network constructed by mining PubMed abstracts
Source: BMC Bioinformatics. 2004 Oct 8;5:147. doi: 10.1186/1471-2105-5-147 (PMC528731; doi:10.1186/1471-2105-5-147)
Supplement: Additional File 2 — The original results of the above study (non-essential files are deleted to keep the file size under the limit set by BMC bioinformatics). [file 1471-2105-5-147-S2.bz2 › chilibotAdditionalFile2/dip05/648ID7922031E11035/html/CGA_CGB.html]

 


 **CGA** and **CGB** 
  
Found 505 abstracts in PubMed, retrieved 05.  
 

 What does Google say? 
 PDF only 
| .edu only 

---

**Interactive relationship** (e.g. stimulation, inhibition, etc)

**Neutral relationship**- In adult islets, alpha cell granules stored  **CgA**  in the halo and  **CgB**  in both the core and the halo, beta cell granules stored both  **CgA**  and  **CgB**  in their cores, and in delta cell granules, both substances were mixed throughout the matrix.  Ref: 12826904 Pancreas, 2003

**Non-interactive relationship** (e.g. studied together, co-existance, homology, etc.)

- The chromogranin family includes chromogranins A  **CgA**  and B  **CgB**  and secretogranin II SgII, once called chromogranin C.  Ref: 12746559 Endocr Pathol, 2003
- INTRODUCTION AND AIMS To get more insight into the differentiation patterns of pancreatic islet neuroendocrine cells and granules during ontogeny, the expression and localization of chromogranin A  **CgA** , chromogranin B  **CgB** , synaptophysin, and insulin were ultrastructurally studied with the immunogold technique in porcine and human pancreatic islet neuroendocrine cells.METHODOLOGY AND RESULTS In porcine pancreas at early fetal stage,  **CgA**  was visualized throughout the immature granules in all neuroendocrine cells.  Ref: 12826904 Pancreas, 2003
- The immunoreactive patterns of  **CgA**  and  **CgB**  were investigated in CJD and compared to those observed in Alzheimer s disease.  Ref: 12692477 Neuroreport, 2003
- We found that  **CgB** , but not  **CgA** , immunoreactivity was selectively associated with prion protein deposits.  Ref: 12692477 Neuroreport, 2003
- new biologic activities for  **CgA** ,  **CgB** , and SgII derived peptides.  Ref: 12746559 Endocr Pathol, 2003
- Recent advances in chromogranin research include establishing the role of  **CgA**  in granulogenesis and the role of  **CgB**  in nuclear transcription.  Ref: 12746559 Endocr Pathol, 2003
- Insulin was expressed in early fetal cells of both pigs and humans, and colocalization with  **CgA** ,  **CgB** , and synaptophysin was demonstrated.CONCLUSION The early expression of  **CgA**  and synaptophysin may reflect a role at early fetal stages, and the individual localization of  **CgA**  and  **CgB**  upon differentiation indicates individual functions.  Ref: 12826904 Pancreas, 2003
- The patient declined surveillance with serial beta human chorionic gonadotropin beta  [ **CGB** ]  hCG  [ **CGA** ]  levels and was lost to follow up.  Ref: 12738129 Obstet Gynecol, 2003
